# SARS-COV-2 – the pandemic of the XXI century, clinical manifestations – neurological implications

**DOI:** 10.25122/jml-2020-0151

**Published:** 2022-03

**Authors:** Vicentiu Saceleanu, Mihai-Stelian Moreanu, Razvan-Adrian Covache-Busuioc, Aurel George Mohan, Alexandru-Vlad Ciurea

**Affiliations:** 1. Department of Neurosurgery, Faculty of Medicine, Lucian Blaga University, Sibiu, Romania; 2. Department of Neurosurgery, County Emergency Hospital, Sibiu, Romania; 3. Department of Neurosurgery, Carol Davila University of Medicine and Pharmacy, Bucharest, Romania; 4. Department of Neurosurgery, Faculty of Medicine and Pharmacy, University of Oradea, Oradea, Romania; 5. Department of Neurosurgery, County Emergency Hospital, Oradea, Romania; 6. Department of Neurosurgery, Sanador Clinical Hospital, Bucharest, Romania

**Keywords:** neurological manifestations, coronavirus, pandemic, CoV – Coronavirus, SARS – Severe acute respiratory syndrome, MERS – Middle East respiratory syndrome, RT-PCR – Reverse Transcription Polymerase Chain Reaction, RBM – RNA binding motif, R0 – Basic reproduction number, HFNO – High-flow Nasal Oxygen, CQ – Chloroquine, CP – Convalescent Plasma, EMA – European Medicines Agency

## Abstract

In December 2019, in Wuhan, China, the first cases of infection with SARS-CoV 2 responsible for COVID-19 disease were identified. SARS-CoV 2 was declared a pandemic on March 11, 2020, and since then has attracted the medical world's attention. The threat to humans' health that this emerging pandemic could leave raises awareness on the importance of understanding the mechanisms that underlie the developing conditions. The epidemiology, clinical picture, and pathogenesis of COVID-19 show that this virus presents new strategies to overcome the past defensive medicine. While all the current data has focused on the pulmonary and cardiovascular manifestations, little has been written about the neurological implications of the disease. This review updates new clinical aspects that SARS-CoV 2 expresses in humans by focusing primarily on neurological manifestations. The damage to the nervous system became more apparent - anosmia, ageusia, polyneuritis, meningitis, meningoencephalitis, stroke, acute necrotizing encephalopathy. Oxygen therapy is vital for those in critical health situations. Finally, prevention is the most important element in breaking the epidemiological chain.

## INTRODUCTION

### General concepts about SARS-CoV-2

Coronaviruses (CoVs) are positive-stranded RNA viruses with spike glycoproteins on the envelope similar to crowns-like shape. The Coronaviridae family includes two subfamilies Torovirinae and Coronavirinae. While Torovirinaes have mostly affected mammals, causing gastroenteritis, the latter predominantly affects the respiratory system. Coronavirinae divides into 4 genera: Alphacoronavirus, Betacoronavirus, Deltacoronavirus and Gammacoronavirus. Among them, 229E, OC43, NL63, and HKU1 strains result in common cold symptoms, while SARS-CoV, MERS, SARS-CoV 2 have caused epidemics and severe respiratory damage [1].

For a long time, CoVs were considered inoffensive to humans causing mild infection symptoms, with the possibility of reinfection dangerous to animals. Literature reported some important damages in animals, such as the porcine epidemic diarrhea CoV (the 1980s) or feline coronavirus infection [2].

However, the outbreak of SARS-CoV in 2002 disclosed the appearance of a new CoV more pathogenic to humans. That contagious pneumonia that appeared in Guangdong in November 2002 at animal handlers rapidly spread across China, Singapore, and Canada by person-to-person transmission. A number of 8422 people were infected in 32 countries, and 919 died [3]. At 10 years' difference, in June 2012, a novel coronavirus was identified in the Arabic Peninsula and was named MERS-CoV based on the dynamic spread. Sequencing the genome reported an evident similarity with bat species from Africa and Eurasia, also belonging to Betacoronavirus genus (as SARS-CoV) but different in lineage type [4].

Despite this similarity with bats, MERS' host and reservoir remain unclear. While sheep, cattle, and bats were tested seronegative for MERS-CoV, a high probability would be that dromedaries and alpacas may be the primary reservoirs for human infection [5]. Until January 2017, 1879 cases were laboratory-confirmed, and 659 deaths were reported by WHO. The overall case fatality rate is ~35%, higher than the previous CoV epidemic in China (~9%). Moreover, MERS persisted for 4 years while the SARS-CoV epidemic for nine months [6].

### SARS-CoV-2 Pandemic

In December 2019, in Wuhan, China, an outbreak of unexplained low-respiratory infection failure was reported to the WHO Country Office, most of the patients having contact with a large seafood market. The situation dramatically and rapidly evolved, the number of people infected with the novel CoV reaching hundreds and thousands. Having the previous experience with CoV epidemics (2002), authorities' response was prompt: sanitation methods, social distancing, passenger screenings, travel hindering [7].

**Figure 1.**
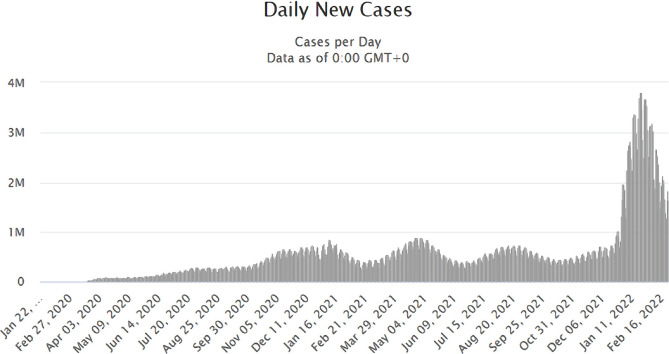
Evolution of the total number of cases registered worldwide until January 27, 2022. Source: https://www.worldometers.info/coronavirus/worldwide-graphs/

**Figure 2.**
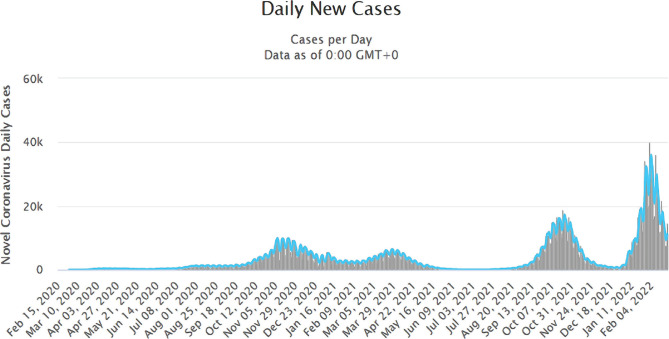
Evolution of the total number of cases in Romania until February 24, 2022. Source: https://www.worldometers.info/coronavirus/country/romania/

On March 11, 2020, WHO declared SARS-CoV 2 outbreak a global pandemic. Until today, there were reported 430,411,767 cases of COVID-19 with 5,938,320 deaths in 216 countries [8] (Figure 1). Until February 24, a total number of 2,697,566 cases were reported in Romania, exceeding the threshold of 1000 cases per day on July 18, 2020 (Figure 2).

SARS-CoV-2 virulence was recently described in 4 stages: invasion, primary blockade of innate antiviral immunity, upregulation of virus protection mechanisms against the human adaptive immunity, and acute and long-term complications of COVID-19 [9].

In the first stage, SARS-CoV 2 penetration into the cells is mediated via the spike glycoproteins localized on the viral envelope. Cell penetration requires two steps: binding and proteolysis, followed by fusion. Both steps are mediated by different subunits S1 and S2, that belong to the envelope spikes (S). Depending on the type of coronaviruses, different domains of S1 are used to localize and bind to the receptor area [10].

Secondly, glycoprotein spikes play an important role in neutralizing antibodies and are the target of the vaccines.Viral priming is based on the activity of some important enzymes that constitute the target for numerous antivirals, such as the endosomal cysteine protease cathepsin L, the serine proteases furin, and TMPRSS2 that are responsible for protein cleavage at spikes site. Residues of amino acids required for receptor ACE 2 binding present in SARS-CoV were conserved into the structure of SARS-S-2. Moreover, even without the expression of DPP4 required for MERS-CoV infection, SARS-S-2 could penetrate the cellular membrane [11].

The third stage includes virus protection from factors of adaptive immunity and generalization of SARS-CoV-2 in the body, while in the late stage, acute onset of the disease with complications are present.

Genomic sequencing showed a 75–80% genomic similitude with the first SARS-CoV due to the errors made by RNA-dependent RNA-polymerase, which made mutations more frequent. However, MERS CoV has not mutated so much overtime to cause an enhancement in infectivity [12]. Specific to SARS-CoV 2 is the similitude with bat RaTG13 which rises up to 98%; the main difference is the furin binding site "RRAR" involved in the cellular priming [13]. Another study reported similar bat-derived coronavirus strains: bat-SL-CoVZC45 and bat-SL-CoVZXC21 [14].

In order to understand the structural basis of receptor recognition, the SARS-S-2-RBM-ACE 2 complex was crystallized. Similar to SARS-CoV, SARS-S-2 forms a concave surface with a ridge on one side. Unlike the SARS-CoV, SARS-S-2 establishes more and stronger contacts with the ACE 2 binding site and has a large contact surface.

That is how the high contagion is explained [12]. In addition, the conformation of the loops in the ACE 2 –binding ridge differs. That is why an additional hydrogen link is presently leading to a more compact conformation between receptor binding motifs (RBM) loops and receptor ACE 2 [15].

### Transmissibility and contagion

Basic reproductive rate (R0) is the metric used to describe transmissibility, defined as the average number of secondary transmissions. R0 depends more on the closed groups or family clusters than community transmission. SARS-CoV R0 was between 2.4 in the first epidemic period, with a downfall after authorities' measures were introduced. For MERS, R0 was 0.69, and for 2009 Influenza A H1N1, R0 was 1.7. However, for SARS-CoV 2, R0 is considered to be 2.5 [16].

Symptoms of the new coronavirus are milder than the past SARS-CoV and MERS, but it is transmitted faster than the other two. Also, the mortality rate is lower (3.4%) than SARS-CoV (9.6) and MERS (35%). Downregulation of IFN-γ and interleukin presented in a patient with comorbidities (diabetes) has been linked to a reduction in the immune response [17].

### Diagnosis

Covid-19 is clinically diagnostic based on fever, fatigue, myalgia, chills and respiratory symptoms, ground-glass opacity chest radiography. In severe cases, patients develop dyspnea one week after the onset of symptoms and evolve rapidly to acute respiratory distress syndrome (ARDS). [18] Nasal or pharyngeal exudate, bronchoalveolar lavage fluid, or bronchial aspirates could be used to identify viral genome via RT-PCR assay. Otherwise, an antibody titer SARS-CoV-2 IgG increased 4 times in the recovery period compared to the acute phase is specific for SARS-CoV 2 infection [19]. Antibody detections are possible from day 4 onward. The difference of antibody response between mild and severe cases is statistically significant from day 15 onward, with bolder antibody response in severe cases. Also, the IgM antibody peak is faster in mild patients [20].

Even though the golden standard for COVID-19 diagnosis is RT-PCR, pulmonary CT could be a complementary tool, especially when false-negative patients have presented symptoms for more than a few days. Also, it has been demonstrated that CT sensitivity (97%) is higher than Rt-PCRCT (83%), especially in the first days [21]. CT shows ground-glass opacity with subpleural distribution and multiple lobes, especially the inferior lobes. A high value of procalcitonin has been linked to worse disease evolution. The possible explanation is that procalcitonin synthesis is stimulated by IL and tumor necrosis factor (TNF)-α and is inhibited by INF-γ [22].

### Pathogenesis

The pathogenic mechanism of producing severe pneumonia is still debatable. The most probable cause is an excessive immune reaction, also called "cytokine storm", but hypoxia, immobilization, and intravascular coagulation contribute to vascular damage [23, 24]. This mixture of modulatory substances is composed of high-levels of IL-1B, IFNγ, CXCL10, and CCL2 responsible for activating T-helper lymphocytes (Th1), leading to an abnormal attack of the organism causing respiratory insufficiency and multiple organ failure. Among the most important interleukin, IL-6 is responsible for the direct activation of T-helper 17 cells, and increased levels have been associated with infection severity [25].

Clinically, ARDS results from an inflammatory injury to the alveolo-capillary membrane, resulting in an increase in alveoli and an accumulation of a proteinaceous liquid in the airspace, provoking hypoxemia and dyspnea [26]. Evidence was found on the direct infection of the endothelial cells at the capillary level causing diffuse endothelial inflammation. Consequently, endothelial dysfunction leads to vascular dysfunction associated with thrombosis, tissular necrosis, and autoimmune response [27]. The term used is "Micro CLOTS" and describes an obstructive thromboinflammatory syndrome [28]. In such situations, clot formation is extremely rapid and highly resistant. While increased levels of fibrinolysis inhibitors may cause this prevailing thrombosis, some results sustain complement (C5a, C5b-9) involvement in the clot formation [29].

Based on several observations, doctors have concluded that among COVID-19 patients, there could be two pneumonia phenotypes (type H and type L) which is an interaction between several factors such as infection severity, patient response to hypoxemia, and time elapsed between onset of the disease and start of medical care [30]. Phenotype L is characterized by low elastance or high compliance; consequently, the amount of gas in their lungs is almost normal, and their evolution is asymptomatic for a while. However, in the presence of pulmonary stress, their medical situation could be worsened and evolve to phenotype H, which is characterized by increased edema, high elastance, and decreased air gas volume.

### Neurological Manifestations

Symptoms may vary from mild manifestations that do not require specialized medical treatment to severe forms of pneumonia – SARS. The highest risk of severe clinical infection is present in older patients (>65 years) and those with underlying pathologies [31].

Unlike MERS and SARS, COVID-19 tends to cause lower respiratory tract infections and rare upper respiratory tract manifestations (sore throat, rhinorrhea).

At the same time, both MERS, SARS, and COVID19 affect the cardiovascular system [32]. Numerous neurological manifestations were reported among the symptoms of COVID19 infection, the most common being headache, dizziness, ataxia, acute cerebrovascular disease, seizures, and acute encephalomyelitis [33].

Headache is one of the most present symptoms of COVID19, with a rate from 6.3% to 23%, depending on the group analyzed [34]. In a study by Wang *et al.*, of 138 hospitalized patients with COVID19, 13 had dizziness, and 9 had headaches. Also, patients admitted to intensive care are more likely to have headaches [35].

A recent study in China, conducted on more than 200 participants, demonstrates the presence of nervous system pathologies in people infected with SARS-CoV-2. These include acute cerebrovascular disease, loss of taste and smell, visual disturbances, and muscle neuralgia, thus indicating impairment of the central and peripheral nervous systems. These manifestations were observed in patients with severe clinical forms [36].

Another study conducted by the same group, in China, on 219 patients infected with COVID-19 shows the appearance of cerebrovascular symptomatology, such as cerebral venous sinus thrombosis, cerebral hemorrhage, ischemic stroke. These manifestations were developed by elderly patients (>75 years) who presented severe clinical forms [37].

Consistent with studies in China, neurological pathological manifestations were reported in a study conducted in France on 58 patients infected with SARS-CoV-2 in severe clinical form. 48 (84%) of them developed nervous system disorders. The neurological manifestations developed include encephalopathy, corticospinal tract dysfunction, delirium, and agitation. Using magnetic resonance imaging in a small group of patients, meningitis-like leptomeninges were observed, and 2 patients had small ischemic strokes [38].

Confusion or loss of consciousness can be caused by intracranial hemorrhage; a recent case in Iran shows massive intracerebral hemorrhage of a COVID-19 patient [39]. Similar to COVID19, clinical cases of neurological pathologies such as seizures and myopathy have been reported during the SARS epidemic [40, 41]. In another study involving 206 patients infected with SARS, 5 (2.42%) cases of acute cerebrovascular disease were observed [42].

During the epidemic of MERS, there have been cases of neurological manifestations, such as neuropathy, delirium, and acute cerebrovascular disease. For example, in a study conducted on 70 MERS patients, 6 cases of seizure and 18 cases of confusion were reported [43, 44].

MERS-CoV infects cells by binding to the dipeptidyl peptidase 4 receptor (DPP4), while SARS-CoV enters cells by binding to the angiotensin 2 converting enzyme (ACE2) receptor. ACE2 is a protein bound to the cell membrane, expressed on the surface of various tissues, including the brain [45, 46].

DPP4 receptors are mainly distributed in the mucous of the lower respiratory tract, kidneys, small intestines, liver, and immune system cells. ACE2 receptors are found on the surface of the cells of the respiratory mucosa, lung parenchyma, vascular endothelium, kidneys, and small intestine [46, 47].

The involvement of the ACE2 receptor in the penetration of SARS-CoV-2 into cells has recently been demonstrated [48]. Similar to SARS-CoV, SARS-CoV-2 attaches to the host cell by binding virion-encapsulated glycoproteins to ACE2 receptors [48]. The affinity of SARS-CoV-2 glycoprotein spikes for ACE2 receptors is higher than that of SARS-CoV for ACE2 receptors [13].

However, the mere presence of ACE2 or DPP4 receptors is insufficient to make a cell susceptible to infection. For example, some endothelial and small intestinal mucosa cells with ACE2 receptors on the surface of the cell membrane were not infected with SARS-CoV following exposure [50, 51].

Another study shows the possibility of infecting cells with low ACE2 receptors, such as hepatocytes, with SARS-CoV [52]. In the same way, cases of MERS-CoV and SARS-CoV infection have been reported in the central nervous system, where the abundance of DDP4 and ACE2 receptors is very low under normal conditions [53].

In 2002–2003, during the SARS epidemic, autopsies of deceased patients infected with SARS-CoV were collected, and the viral particles detected were located almost exclusively at the level of neurons. [54, 55]. The exact route by which coronaviruses reach the central nervous system is still unknown. The hematogenous or lymphatic pathway seems possible, especially in the early stages of infection, given that almost no viral particles were detected in nonneuronal cells in the affected brain areas. [54–56]. Another possible route is represented by coronavirus infection of the peripheral nervous system, peripheral nerve endings, with viral particles reaching the central nervous system through a synaptic route [57–59]. Experimental studies in transgenic mice in which SARS or MERS viral particles were administered intranasally showed the possibility of virions penetrating the brain through olfactory nerves followed by the rapid spread of infectious entities to specific brains, including the thalamus and brainstem [49, 60]. Another possible route may be via infected leukocyte migration, which may play the role of a "Trojan horse", passing through the blood-brain barrier [61].

Anosmia and ageusia are the most common peripheral nervous system disorders caused by SARS-CoV-2, reported in the symptoms of infection and other coronaviruses in humans. Anosmia begins suddenly, usually with a few additional symptoms, such as nasal obstruction or rhinorrhea [62].

Anosmia and ageusia are present mainly in asymptomatic patients or during manifestations of infection with the new coronavirus [63]. Thus, people who are asymptomatic but have a loss of taste and smell are, by default, carriers of SARS-CoV-2, having the ability to spread the virus. Most patients gradually recover their taste and smell as they recover from SARS-CoV-2 infection [64]. The exact mechanism of developing anosmia due to infection with the new coronavirus is not yet fully understood. An animal study demonstrated the ability of coronavirus to spread transneuronally until it reaches the brain via the olfactory pathway while affecting the integrity of the olfactory neuroepithelium by binding to TMPRSS2 and ACE2 receptors on the membrane of supporting cells [65, 66]. However, this early speculation has been dismantled since a recent analysis of the CSF from living patients with neurological manifestations failed to confirm the viral presence. Viral RNA was barely seen in the CSF of patients who died of COVID-19 [67]. On the other hand, studies have demonstrated increased inflammation within brain parenchyma and CSF. CSF in COVID-19 patients exhibits an expansion of dedifferentiated monocytes and exhausted CD4+ T cells, with a lower interferon level than in viral encephalitis [68]. In the CSF of individuals with COVID-19, dendritic cells transcriptional profile was regulated; most of these genes were classified as type 1 and type 2 interferon-stimulated genes. Interleukin 1 (IL-1) and IL-12 were increased in CSF compared to the blood samples. Genes associated with natural killer (NK) cell activation were also stimulated in the CSF COVID-19 patients [69].

Also, neurological manifestations were observed in the 2002 SARS epidemic. SARS-CoV-1 was detected using the PCR technique in the cerebrospinal fluid of a 32-year-old woman with tonic-clonic seizures [70]. A 59-year-old woman with IgA nephropathy and SARS also had encephalopathy and seizures [71].

Cases of encephalitis and COVID19 have been reported, but one case of acute necrotizing encephalopathy was reported in a 50-year-old woman with dry cough, fever, and impaired cognitive ability. Following magnetic resonance imaging investigations, a cerebral hemorrhage was observed, affecting both the thalamus and the medial region of the temporal lobe and the subinsular regions [72].

Secondary to inflammation and oxidative stress, hippocampal and cortical atrophy may appear, small vessel disease, and hypoxic-ischemic changes. Diffuse moderate to severe brain edema was seen as a hypoxic change. Post-mortem staining indicated a loss of neurons in the cerebral cortex and hippocampus and a high amount of infiltration surrounding vascular structures along with parenchymal nodules. In a semi-quantitative analysis, microglial activation was increased especially in the brainstem (pons), and hippocampus microglial activation was significantly increased in cases with delirium [73].

The pathophysiology of COVID-19 may be responsible for an increased risk of stroke. Severe clinical cases were observed in elderly patients, who generally had comorbidities that increased the risk of stroke. As with SARS infection, the onset of hypercoagulability syndrome may complicate the clinical condition of a COVID-19 patient [40].

There is a temporal correlation between stroke onset and the peak of acute phase reactants (CRP, ferritin, D-Dimer). COVID-19 patients with stroke showed elevated levels of endothelial activation markers (von Willebrand activity, von Willebrand antigen, factor VIII). In addition, higher levels of IL-6 and sIL-2R were noted in COVID-19 patients. This evidence suggests endotheliopathy and systemic inflammation are more frequent in COVID-19 patients leading to ischemic stroke in patients with vascular risk factors compared to control groups [74].

Merkler *et al.* found a relatively increased frequency of acute ischemic stroke in severe COVID-19 patients (31/1916; 1.6%) compared to patients with influenza (3/1486; 0.2%) [75].

The abundance of coagulation markers may be increased in SARS-CoV-2 infection, and disseminated intravascular coagulation has also been observed [76]. It has been observed that viral myocarditis, a subsequent infection with the new coronavirus, may increase the risk of stroke [77].

Cells in the vascular endothelium of arteries and veins in the brain have ACE2 receptors on their surface, with a risk of developing viral-induced vasculitis. The risk increases for arterial and venous diseases. In a study by Mao *et al.*, in 78 COVID-19 patients, stroke was observed in 6 cases (2.8%), 5 ischemic, and 1 hemorrhagic [78]. In a smaller study, stroke was observed in 2 cases (8%) of 24 COVID-19 patients [79].

The autonomic nervous system (ANS) is also affected by COVID-19. Orthostatic hypotension and postural tachycardia syndrome (POTS) may be impaired. Sudomotor, gastrointestinal and pupillomotor dysfunction are also encountered. The time of the visit suggests minor differences in the spectrum of complications, but females seem to be affected. Overall, ¼ patients suffered from "global dysautonomia" [80].

Neurological events were encountered even after the COVID-19 vaccination, even though at a minuscule rate compared to the enormous number of patients. Despite the common symptoms such as fever, myalgia, fatigue, and arthralgia, some of these manifestations post-vaccination were cerebral venous sinus thrombosis, Bell's palsy, acute transverse myelitis, and acute disseminated encephalomyelitis that may occur due to the phenomenon of molecular mimicry [81].

Observing the evolution of the pandemic, especially the symptoms developed following the SARS-CoV-2 infection, we cannot say that neurological disorders are extraordinarily common. The most common neurological manifestations of the new coronavirus infection are headache, loss of taste and smell, and dizziness; diseases of a cerebrovascular nature affecting a small number of patients, most of them with advanced age and underlying pathologies.

### Treatment and Prevention. Vaccination

For patients with respiratory impairment, the first step is oxygen therapy. The goal is to maintain the oxygen saturation >90% and >92–95% for pregnant women. Nasal cannula, simple masks, or venturi masks are used to deliver oxygen. High-flow oxygen systems (HFNO) provide high-oxygen concentrated air to patients and are associated with a low risk of subsequent intubation and shorter hospitality stay [82]. HFNO generates a decrease in respiratory resistance and a positive end-expiratory pressure while decreasing the need for an invasive respiratory supplement, yet the risk of airborne transmission is still debatable [83, 84].

Molecularly, effective oxygen therapy reduces hypoxia responsible for increased ACE 2 expression, thus leading to a less aggressive virus manifestation in an organism. Nocturnal oxygen therapy is speculated to delay the progression of SARS-CoV 2 replication [85].

Administration of corticosteroids is considered to be a sword with two edges. Among patients receiving air support, dexamethasone was demonstrated to reduce 28-days mortality by one-third [87]. Another study reported that administration of methylprednisolone to severe patients reduced the risk of death by 62% [87]. Corticosteroids induce immune suppression, antagonizing inflammation and reducing cytokines [88].

Regarding antiviral therapy, the classic lopinavir with ritonavir combination used so far in HIV treatment seemed to be inefficient against SARS-CoV 2, showing no benefits at all [89].

Moreover, it may be possible for a new triple combination of antivirals to be efficient in the fight against this novel virus, specifically: interferon beta-1b, lopinavir-ritonavir, and ribavirin. The patient from the triple combination group reported a shorter period of hospital stay and a significant reduction in nucleic acid positivity than in the control group. Side-effects were limited to liver affection (14%)[90].

Other drug combinations that showed potential effective results were: Sirolimus plus Dactinomycin (which may inhibit mTOR signaling and RNA synthesis), Toremifene plus Emodin, Mercaptopurine plus Melatonin (which play an important role in ACE 2 regulation in SARS-CoV and is now also tested for SARS-CoV 2) [91].

Chloroquine (CQ) and its derivatives used so far for anti-malaria effects were recently effective against SARS-CoV 2. Studies reported a decrease in hospitalization and faster negative Rt-PCR conversion in patients treated with CQ or derivatives [92, 93]. On the other hand, other studies showed that the hydroxychloroquine (HCQ) group had no difference in ventilation dependence or mortality. Moreover, patients administered HCQ presented long QT intervals and a higher risk for developing "torsades des pointes" [94]. In the absence of a bacterial infection, antibiotics should be avoided in patients with SARS-CoV 2 [95].

Convalescent plasma (CP) is defined as the immunotherapy during which patients recovered after viral infection donates plasma with high-antibody titre to treat infected people. Patients treated with a dose of 200 mL CP experienced symptom improvement, increasing the lymphocytes count, decreasing C-reactive protein, and 70% of the patients got rid of the viral load [96]. Even though there have been small studies including CP so far, this therapy may be a promising tool in the future.

The European Medicines Agency (EMA) approved 5 vaccines against COVID-19, and more than 10 billion doses have been administered globally [97]. COVID-19 vaccines are produced under different mechanisms. These include the usage of subunits or recombinant antigen proteins with a weak immunogenic effect and generally need an adjuvant to potentiate their effect. The most common recombinant vaccine proteins are spike N and S proteins. Protein N covers the viral genome and is responsible for releasing virions, while protein S plays a role in binding to the host cell via the amino acid chain [98].

Viral vector vaccines are effective solutions for transmitting viral genes and provide a long-lasting synthesis of antigenic proteins which are not consumed as quickly as subunit proteins. mRNA-based vaccines – BNT162b2 (Pfizer-BioNTech, New York, New York) and mRNA-1273 (Moderna, Inc., Cambridge, Massachusetts), comprise the mRNA sequence in a lipid nanoparticle expression gene for a viral protein. Anti-vector immunity is barely seen since the immunogenic reaction of the mRNA sequence is weak [99].

At the beginning of 2021, the third vaccine was approved in the European Union produced by AstraZeneca/Oxford University. The ChAdOx1 vaccine is based on an adenoviral vector from the chimpanzee containing the spike glycoprotein transcription gene [100]. The vaccine is 70.4% effective after both doses and 64.1% after a single standard dose against symptomatic disease. Large differences in vaccine efficacy depending on the outcomes of each population group (UK, Brazil) were not reported (60.3% and 64.2%, respectively). Although the vaccines were given at different periods of 6 weeks (Brazil) and 12 weeks (UK), it did not cause a significant difference in the number of antibodies developed [101]. AstraZeneca vaccine side effects were an open debate table. Studies reported mild symptoms such as tiredness in 52.08–53.1% cases, headaches for healthcare workers in 50.15–52.6% cases, and myalgia in 42–44.0% cases [102, 103]. However, reports of clotting issues (thrombosis in combination with thrombocytopenia) lead many European countries to temporarily stop the administration of certain vaccine batches. This type of thrombosis was also reported after the Johnson & Johnson/Janssen vaccination, which resembles autoimmune heparin-induced thrombocytopenia, a condition that is triggered when heparin binds to a protein called platelet factor 4 and stimulates the production of antibodies against platelet factor 4, which eventually leads to platelet destruction and the release of clot-promoting material [104, 105]. However, most of them resumed vaccination after the European Medicines Agency (EMA) declared that the benefits of AstraZeneca vaccination outweigh its risks.

Moderna Vaccine Inc. Cambridge MA, USA, was introduced in 2021 as the first mRNA vaccine (mRNA-1273) used against SARS-CoV-2. The vaccine includes mRNA lipid nanoparticle structures for S protein synthesis and has set a record time from its production to widespread use of only 63 days. In the 3^rd^ phase of testing, the vaccine was 94.1% effective, with 3.3 out of 1000 people being infected with COVID-19 after the vaccine, while in the placebo group, the infection rate was much higher at 56 out of 1000 people [106]. Immunogenicity of the vaccine was reported 119 days after the first vaccination dose and 90 days after the second dose (100 μg), the two doses being administered 28 days apart [107].

The BNT162b2 vaccine (Pfizer/BioNTech) was introduced in December 2020 in Romania. It contains lipid nanoparticles corresponding to a modified mRNA encoding a spike protein. Side effects of the vaccine include mild to moderate short-term pain at the vaccination site, with headache, myalgia, and asthenia. Immunity after the second dose of vaccination is 95%, for a minimum of 2 months' characteristic of other types of viral vaccines. Doses of 30 μg are administered every 21 days. Compared to age samples, young people had higher immunity from vaccination than the elderly [108]. Although the effectiveness of the vaccine after the first dose is reported at 52.4%, it is considered that it could be much higher in reality, reaching around 92.6%. Booster efficacy was tested and reported in the Israeli population (one of the most immunized populations) in the elder population. They found that at least 12 days after the booster dose, the number of positive cases was lower in the booster group than in the non-booster group by a factor of 11.3, and the rate of severe illness was lower by a factor of 19.5. Thus, booster administration efficacy is substantially high and should be considered, especially in vulnerable groups [109].

Another 2 vaccines are to be used, namely, the one produced by Johnson & Johnson based on a human adenoviral vector Ad26, whose antibody titer remains stable until day 71, and the vaccine NVX-CoV2373 (Novavax). On January 29, 2021, Janssen announced that the vaccine was 66% effective against COVID-19 and 85% against the development of a severe form of COVID-19 [110].

The Johnson & Johnson vaccine was approved on March 11, 2021, and became the fourth vaccine used in the European Union. During April 13–23, 2021, CDC and FDA recommended temporarily stopping the use of Janssen vaccine after 6 reported cases of cerebral venous sinus thrombosis similar to those reported after the administration of the AstraZeneca vaccine in Europe. Among the reviewed reports, 97% were classified as non serious and 3% as serious manifestations, including thrombosis in large arteries or veins, accompanied by thrombocytopenia [111]. The advantages of this vaccine are the administration of a single dose as opposed to the other three vaccines already approved in the EU, which require the administration of two doses.

Novavax is a subunit vaccine that comprises the recombinant protein S in a saponin-based M-matrix that binds to the ACE2 receptor. This vaccine triggers a strong immune response with specific anti-protein S antibodies, neutralizing the effects of the virus [112]. Novavax became the 5^th^ vaccine against COVID-19 to be used in Europe.

## CONCLUSIONS

The 3^rd^ wave of coronavirus extended globally, characterized by contagiousness and high morbidity. The main lesions caused by coronavirus were found in the lungs, cardiovascular system, renal and digestive systems. Being an endothelial disease, COVID-19 rapidly produces multiple organ dysfunction syndromes (MODS) with an extremely severe prognosis in some cases.

Neurological alterations have been mentioned sporadically but are present, ranging from anosmia and ageusia to multiple central and peripheral nervous system damages. The significant neurological lesions so far identified both clinically and neuroimaging certainly consist of meningoencephalitis, encephalitis with marked cerebral edema, acute necrotizing encephalopathy, stroke, Guillain-Barré syndrome. Vaccination is the only effective method to stop virus spread. Treatments include antipyretics, anti-inflammatory drugs, anticoagulants, antivirals, and immune-boosting drugs. Finally, prevention is the most important element in breaking the epidemiological chain.

## ACKNOWLEDGMENTS

### Conflict of interest

The authors declare no conflict of interest.

### Authorship

AVC and MSM contributed to conceptualizing the manuscript. VS, MSM, RACB contributed to the methodology, writing the original draft and editing the manuscript. AGM contributed to data collection and data analysis.
